# Changes in Magnetic Resonance Signal Fluctuation in Superior Sagittal Sinus: Deterioration of Arteriolar Vasomotor Function of Young Smokers

**DOI:** 10.3390/tomography8020055

**Published:** 2022-03-02

**Authors:** Minghui Tang, Masaya Kubota, Yusuke Nitanda, Toru Yamamoto

**Affiliations:** 1 Department of Diagnostic Imaging, Faculty of Medicine and Graduate School of Medicine, Hokkaido University, Sapporo 060-8638, Japan; toumeiki@hs.hokudai.ac.jp; 2 Department of Health Sciences, School of Medicine, Hokkaido University, Sapporo 060-8638, Japan; hypnos_yakumo@eis.hokudai.ac.jp; 3 The 1st Department of Radiology, Kin-ikyo Chuo Hospital, Sapporo 007-8505, Japan; yosakoi.tantan@gmail.com; 4 Division of Biomedical Engineering and Science, Faculty of Health Sciences, Hokkaido University, Sapporo 060-0812, Japan

**Keywords:** MRI, venous blood, arteriolar vasomotor function, smoking

## Abstract

(1) Cerebral arteriolar vasomotor function is vital for brain health and has been examined through CO_2_ inhalation or breath-holding, which are both challenging for patients. We have developed a non-invasive method to evaluate this function with magnetic resonance imaging (MRI) by utilizing respiration-induced natural changes in partial pressure of arterial CO_2_ (PaCO_2_). In this study, we applied this method for 20s to evaluate the chronic effect of a few years smoking on the cerebral arteriolar vasomotor function. (2) A single slice (five slice thicknesses: 15 mm to 7 mm) perpendicular to the superior sagittal sinus of was imaged successively for 45 s using spin-echo echo-planar imaging by 3T MRI for ten smokers (24.5 ± 1.6 years) and ten non-smokers (24.3 ± 1.4 years), respectively. The venous oxygenation fluctuation (Δ*Y_r_*) caused by the respiration-induced changes of PaCO_2_, which reflects the arteriolar vasomotor function, was calculated from the time series MR signal changes of superior sagittal sinus. (3) The Δ*Y_r_* values of the smokers (0.7 ± 0.6) were significantly lower than those of the non-smokers (1.3 ± 0.8) (*p* = 0.04). (4) Degeneration of the cerebral arteriolar vasomotor function due to chronic smoking (even after 20s) was demonstrated by our non-invasive MRI-based method.

## 1. Introduction

Arterioles are small arteries that diverge into the capillary bed. They play a major role in resisting the flow of blood in blood vessels, providing approximately 80% of the total resistance to blood circulation and greatly affecting the blood flow and blood pressure [1]. Regulation of blood flow and intravascular pressure is especially crucial in the brain, and the cerebral arterioles regulate them by vasodilation or vasoconstriction due to changes such as in the partial pressure of arterial carbon dioxide (PaCO_2_), or in mediators such as nitric oxide during high metabolic activities of the parenchyma [1,2]. This capability of vasodilation and vasoconstriction is known as cerebral arteriolar vasomotor function, and it degenerates owing to vasculitis or arteriolosclerosis [1], causing hypoperfusion [3], cerebral ischemia, and augmentation of amyloid-β accumulation, which is a neurotoxic factor [4,5]. In particular, cerebral amyloid angiopathy due to accumulation of amyloid-β results in forms of dementia such as Alzheimer’s disease [6,7]. Therefore, deterioration of the arteriolar vasomotor function is expected to be an early biomarker of dementia [8,9,10]. Cerebral arteriolar vasomotor function is important for brain health and would be useful in diagnosing dementia, as well as predicting the possibility of its occurrence.

The arteriolar vasomotor function has been evaluated by measuring the cerebrovascular reactivity (CVR), such as blood flow changes due to forced PaCO_2_ modulation. The PaCO_2_ level, which is monitored by end-tidal partial pressure of CO_2_ (PetCO_2_) measurement [11], is normally changed by CO_2_ inhalation (CO_2_ administration) or breath-holding. The changes in blood flow velocity due to CO_2_ inhalation were measured using transcranial Doppler ultrasonography (TCD) in the middle cerebral artery, and a decrease in the CVR was observed in patients with severe intracranial hemorrhage [12]. Near-infrared spectroscopy (NIRS), which observes the changes in the concentration of oxyhemoglobin and deoxyhemoglobin in blood, is also sensitive to PaCO_2_ changes caused by CO_2_ inhalation [13]. By setting NIRS probes on the forehead of patients with stenotic disease, oxyhemoglobin was observed to decrease with the severity of stenosis [14]. Although the TCD method measures the blood flow velocity in the middle cerebral artery to evaluate the CVR, whereas the NIRS method can evaluate the local CVR such as in the forebrain, measurement errors that occur due to the contamination of extracranial circulation need to be addressed [14]. To map the arteriolar vasomotor function, positron emission tomography (PET) was used to successfully evaluate the CVR due to CO_2_ inhalation by mapping the changes in cerebral blood flow (CBF) [15]. Furthermore, a radiation-free mapping technique for CVR was developed using magnetic resonance imaging (MRI) with CO_2_ inhalation [16]. Alternatively, breath-holding without CO_2_ inhalation has also been used to change PaCO_2_, and the breath-holding index (BHI) has been introduced. The increase in the blood flow velocity from the resting baseline value due to breath-holding is measured and its percentile increase is divided by the breath-holding time to obtain the BHI [17]. Breath-holding methods have been successfully applied to CVR mapping [18]. Additionally, the visually evoked CBF response, which represents the CBF changes induced by visual stimulation, has been successfully measured using TCD [19].

The abovementioned methods have been used to study the effect of smoking on the arteriolar vasomotor function. Right after smoking, cerebral blood flow velocity in the middle cerebral artery increased but BHI decreased, indicating a decrease in CO_2_-driven vasomotor reactivity [20]. The same tendency was also confirmed by TCD measurement of the middle cerebral artery and NIRS measurement on the forehead with CO_2_ inhalation [21]. These phenomena are understood by pharmacological effect of smoking that dilates arterioles [22], resulting in a decrease of their expandability and hence a decrease of CO_2_-driven vasomotor reactivity [21]. This acute effect of smoking ceased approximately 30 min after smoking. Chronic smoking also affects the arteriolar vasomotor function. It has been reported that CBF decreased gradually at age from 20s to 90s and CBF in smokers was significantly lower than that in non-smokers at all ages [23]. Even in the late young-age smokers (around 30 years old), CBF [24], BHI [25], and visually evoked CBF response [19,26] were lower than those in non-smokers, showing a degeneration of arteriolar vasomotor function due to chronic smoking of approximately 10 years.

Although the aforementioned methods evaluate the arteriolar vasomotor function, there remain a few issues to be addressed for further investigation. First, the methods of CO_2_ administration are invasive and challenging for patients. Second, the breath-holding based methods may have low reproducibility and high variability of BHI values since some patients may be unable to perform breath-holding [27], or the breath-holding activity may be performed inaccurately [28]. Third, the method employing the visually evoked CBF response is based on neurovascular coupling, the decrease in visually evoked CBF response could be caused by either an impaired interaction between neurons/glial cells and endothelium or by an impaired vasodilator ability [26]. A method to measure the arteriolar vasomotor function non-invasively and directly is needed for not only patients but also healthy volunteers. Such a method has the potential to be widely adopted in studies involving a broad range of volunteers.

We focused on the respiratory fluctuation of PaCO_2_ and developed a non-invasive method to evaluate the arteriolar vasomotor function using an MRI without CO_2_ inhalation or breath-holding [29]. Changes in PaCO_2_ causes the vasodilation or vasoconstriction of the arterioles, and hence the respiratory fluctuation of PaCO_2_ varies the cerebral blood flow leading to the fluctuation in oxygenation level in cerebral venous blood. The amplitude of this fluctuation of venous oxygenation at respiratory frequency represents the arteriolar vasomotor function. By analyzing the spontaneous changes in the MR time series signals of the cerebral venous blood, the venous oxygenation fluctuation caused by the respiration-induced fluctuation of PaCO_2_, which reflects the arteriolar vasomotor function, was calculated. In this study, we applied this method to the young population (middle 20s) to evaluate the chronic effect of a few years of smoking on the arteriolar vasomotor function. This method is easy to apply and can be conveniently adopted in large-scale MRI based studies.

## 2. Materials and Methods

### 2.1. Subjescts

Ten smokers (male; 24.5 ± 1.6 years; smoking history 4.6 ± 1.3 years; number of cigarettes per day 11 ± 2) and ten male non-smokers (male; 24.3 ± 1.4 years) participated in this study. All volunteers were in good health. The details of the smoking habits of the smokers are presented in Table 1. The smokers abstained from smoking for more than 8 h before the data acquisition to avoid the influence of carbon monoxide (CO), which is also a vasodilator; 8-h abstention is needed to remove CO from the blood [30]. This study was conducted in accordance with the Declaration of Helsinki and approved by our institutional review board, and informed consent was obtained from all of the volunteers.

### 2.2. Data Acquisition (Tang 2017) 

We used a 3T MRI scanner (Magnetom Prisma, Siemens Healthcare, Erlangen, Germany) with a 64-channel head coil. A single sagittal slice (slice thickness = 10 mm, field of view [FOV] = 240 mm × 240 mm, matrix size = 256 × 256), which included the superior sagittal sinus, was imaged using 2-dimensional (2D) fast low-angle shot (FLASH) phase contrast (PC) imaging synchronized with cardiac pulsation, which was monitored using a built-in fingertip pulse monitor. The cardiac cycle was divided into 20 phases, and the PC image at each phase was obtained. The velocity encoding was set to 400 mm/s. The scan time of this 2D FLASH PC imaging was approximately 5 min, depending on the individual heart rate. The systolic blood velocity of the superior sagittal sinus was mapped and the blood flow continuity along the sagittal sinus was assessed from the mapping.

To obtain the MR time series data of venous blood in the superior sagittal sinus, a single slice perpendicular to the superior sagittal sinus was imaged successively for 45 s using spin-echo echo-planar imaging (SE-EPI) (echo time [TE] = 30 ms, matrix size = 128 × 128, FOV = 170 mm × 170 mm) with 170-ms repetition time (TR) [29]. The slice position with a small change in the blood flow velocity was selected on the blood velocity mapping to make sure so that there was no turbulence of blood flow due to arachnoid granulation that deteriorates the measurement accuracy. The amplitude of MR signal fluctuation due to blood flow change does not depend on the slice thickness in spin-echo imaging whenever the flow void effect does not occur, while that due to oxygenation change increases with an increase in slice thickness. To extract the fluctuation due to blood oxygenation change, the successive imaging was repeated five times by changing the slice thickness by 2 mm from 15 mm to 7 mm. All obtained images were confirmed that there was no flow void effect, which reflects narrow blood vessel and rapid blood flow. During the imaging, the volunteer breathed at 15 breaths per min by synchronizing with a digital metronome displayed on an MRI-compatible screen visible to the volunteer via a mirror on the head coil. Based on this constant rhythm of breathing, the respiratory component of the time series data appeared at 0.25 Hz on the spectrum. However, the heart rate varied individually and its harmonics in the spectrum crossed the Nyquist frequency (NF), which was determined based on the TR of the time series imaging: NF = 1/(2TR). Therefore, some harmonics of the cardiac pulsation component of the time series data folded back on the respiratory frequency range of 0.2–0.3 Hz. To avoid this contamination caused by the harmonics of the cardiac pulsation component folding back into the respiratory component, we set the TR to 170 ms (NF = 2.94 Hz) so that the uppermost fourth harmonic of the cardiac pulsation component folded back above the upper bound of the respiratory frequency range (0.3 Hz). This TR was determined from the folding frequency of the fourth harmonic of the cardiac pulsation component of the standard higher heart rate (84 min^−1^) obtained from the higher one standard deviation of the average heart rate in the supine position (69.1 ± 13.6 min^−1^) [31]. Note that the influence of the fifth and higher harmonics was ignored since their spectral intensity levels were equal to or lower than that of noise. 

The imaging procedure was performed on each volunteer. Additionally, heart rate, mean arterial pressure (MAP), and PetCO_2_ were measured using an electronic sphygmomanometer (ES-H55, Terumo, Tokyo, Japan) and capnography (WEC-7301, NIHON KOHDEN) before and after the scan by laying the volunteer down on a stretcher outside the scanner room while maintaining a 0.25 Hz respiratory rate.

### 2.3. Data Analysis (Tang 2017)

**Figure 1:**
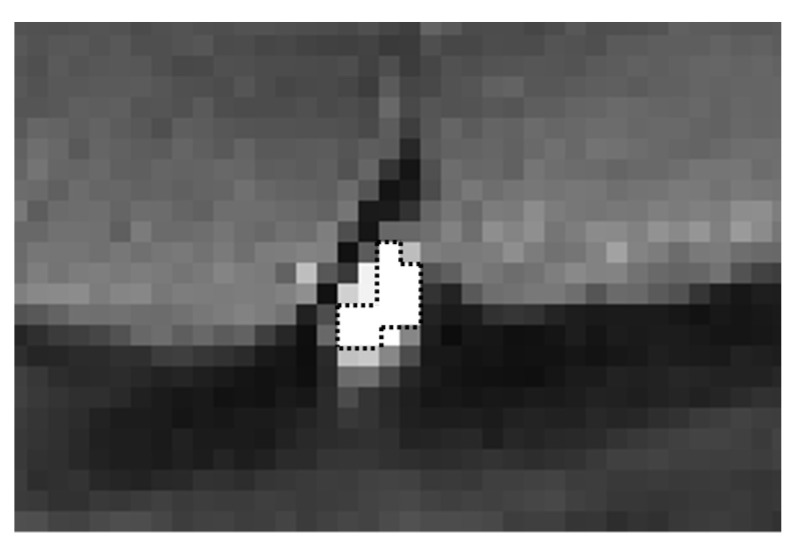
Enlarged view of the superior sagittal sinus of a SE-EPI image. The black dotted line represents the ROI (11 pixels) for the signal measurement in the superior sagittal sinus.

The initial twelve temporal images were excluded from further analysis to allow time for stabilization of the MR signal. The time series of the MR signal images (256 images) was analyzed using MATLAB (R2014b, MathWorks, Inc., Natick, MA, USA). The average signal of 11 pixels with the highest signal intensity (19.4 mm^2^) in the superior sagittal sinus was obtained to represent the MR signal of the superior sagittal sinus on each temporal image (Figure 1). After subtracting the temporal average signal intensity (S), the time series of the changes in the MR signal of the superior sagittal sinus was Fourier-transformed by applying the Hamming window to purify the respiratory component (0.2–0.3 Hz) from the contamination by lower frequency components, which are mainly induced by the default network of the brain function [32]. To confirm the effect of the Hamming window, the values of the power spectral intensity (PSI) at the lowest and highest frequency points of the respiratory component were measured for all spectra of 10 volunteers (five non-smokers and smokers, respectively), and their absolute difference values with and without the Hamming window were compared. The respiratory peak at 0.25 Hz was confirmed to make sure that all volunteers performed the respiratory rhythm correctly following the displayed metronome. The spectral fluctuation intensity at the respiratory frequency (SFIr) was obtained, and its value was multiplied by 1.59, a correction factor of the Hamming window [33]. 

The graph for *SFI_r_* versus *S* was plotted. The slope of the regression line of this plot is expressed by the following equation [29]:

(1) $$\frac{SFI_{r}}{S}=2C\cdot \left(1-Y\right)\cdot TE\cdot \Delta Y_{r}$$

where *C* is a constant determined by the magnetic field strength and hematocrit fraction [34], *Y* is the venous blood oxygenation, and Δ*Y_r_* is the change in *Y* at the respiratory frequency. In this calculation, we used *Y* = 0.66, which is the average venous blood oxygenation in healthy human volunteers [34], and *C* = 59 based on the normal hematocrit (0.46) [35]. The values of Δ*Y_r_* for non-smokers and smokers were obtained from Equation (1) and the slopes of the regression lines (*SFI_r_*/*S*) of all data sets. Welch’s t-test was performed to compare the values of Δ*Y_r_* and the physiological parameters (MAP, PetCO_2_, heart rate) between two groups. The Δ*Y_r_* of smokers versus the Brinkman index (the product of the number of cigarettes per day and duration of smoking) was also plotted.

## 3. Results

**Figure 2:**
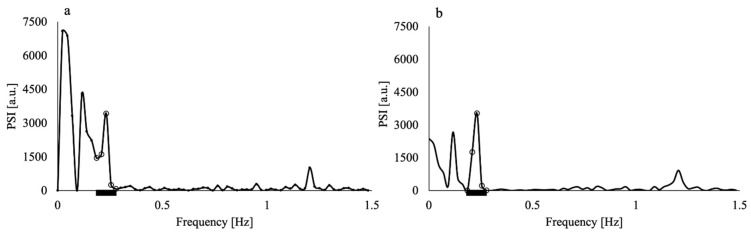
Power spectral intensity (PSI) of the time series MR signal of the superior sagittal sinus of a non-smoker volunteer (**a**) before and (**b**) after application of the Hamming window. The PSI values in (**b**) were multiplied by the square of the correction factor (1.59) of the Hamming window. Both MR signals were obtained through imaging with 15 mm slice thickness. Black thick bars represent the respiratory frequency range. The open circles represent the data in the respiratory frequency range (0.2–0.3 Hz).

**Figure 3:**
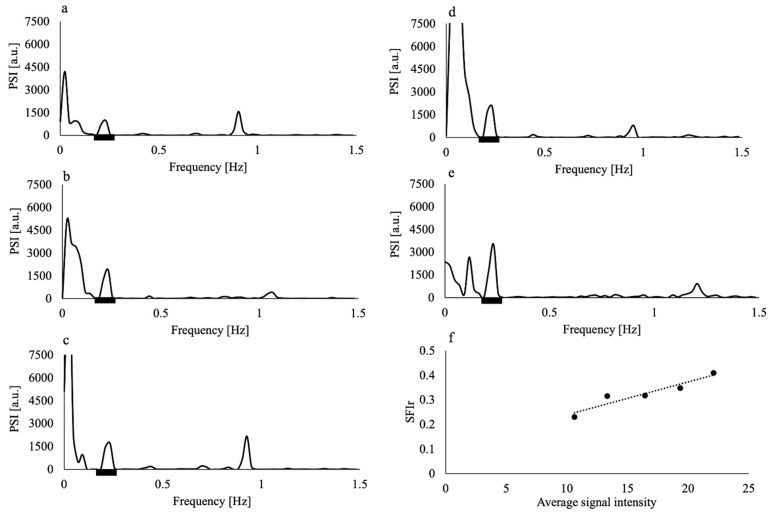
Power spectral intensity (PSI) of the time series MR signal of the superior sagittal sinus from imaging with slice thicknesses of (**a**) 7 mm, (**b**) 9 mm, (**c**) 11 mm, (**d**) 13 mm, and (**e**) 15 mm of a non-smoker volunteer; (**f**) spectral fluctuation intensity at the respiratory frequency (SFIr) versus average signal intensity of the same volunteer. Black thick bars in (**a**–**e**) represent the respiratory frequency range. Dashed line in (**f**) represents the regression line. The data of this volunteer showed the highest coefficient of determination of the regression line (R^2^ = 0.90).

**Figure 4:**
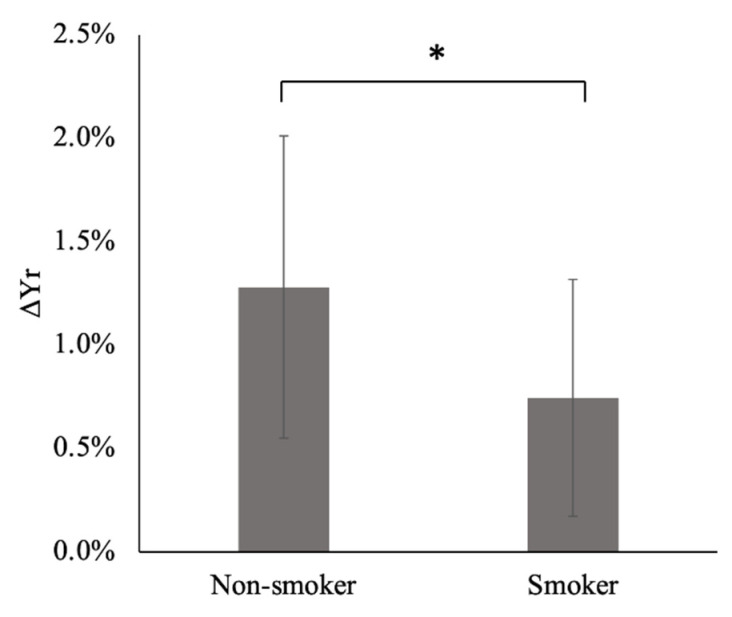
Respiratory changes in venous blood oxygenation (Δ*Y_r_*) of non-smokers and smokers. The error bars indicate the standard deviation. *: *p* < 0.05. See Supplementary Material for further details.

The application of the Hamming window clearly reduced the contamination of the respiratory component by the low-frequency component, as shown in Figure 2; the PSI at the lowest respiratory frequency approached the axis. The absolute difference in the PSI between the lowest and highest frequency points of the respiratory component significantly decreased by approximately half when the Hamming window was applied (*p* = 0.005). The purified respiratory components, which were free of contamination by the low-frequency components, were observed (as shown in Figure 3a–e of all slice results of a non-smoker). These results showed clear linearity of the SFIr versus S plot (Figure 3f). The Δ*Y_r_* values of the smokers were significantly lower than those of non-smokers (*p* = 0.04) (Figure 4). The Brinkman index did not correlate with the Δ*Y_r_* values of the smokers (r = 0.25). The physiological parameters of the volunteers are summarized in Table 2. There was no significant difference in PetCO_2_ (*p* = 0.32), MAP (*p* = 0.47) and heart rate (*p* = 0.10) between non-smokers and smokers. The heart rate of all volunteers was less than the criterion (84 min^−1^) for avoiding the folding of the fourth harmonic of the cardiac pulsation component in the respiratory frequency range. The maximum heart rate was 78 min^−1^.

## 4. Discussion

In this study, we analyzed the spontaneous changes in the MR time series signal of cerebral venous blood and elucidated respiration-induced venous oxygenation fluctuation, which reflects the arteriolar vasomotor function [29], for non-smokers and smokers after preparatory abstention from smoking. Although there was contamination of the respiratory components by the low-frequency components in the spectra of the MR signal fluctuation, this contamination was reduced by applying the Hamming window (Figure 2). The effectiveness of the Hamming window was quantified and confirmed by the significant reduction in the difference in PSI between the lowest and highest values of the respiratory frequency range. As the heart rates of all volunteers were less than 84 min^−1^, the setting of TR = 170 ms prevented the contamination from the harmonics of the cardiac pulsation component. Applying the Hamming window and increasing the Nyquist frequency by using a TR of 170 ms improved the accuracy of Δ*Y_r_*.

The values of Δ*Y_r_* for smokers were significantly (*p* = 0.04) lower than those of non-smokers (Figure 4), demonstrating a degeneration of the arteriolar vasomotor function due to chronic smoking in young smokers, even after a few years of smoking. This result agrees with those of previous studies based on BHI and visually evoked CBF response; the BHI [25] and visually evoked CBF [19,26] decreased in young smokers when compared with non-smokers. Δ*Y_r_* obtained from our spectral analysis of the MR time series signals of cerebral venous blood reflects the arteriolar vasomotor function, and its degeneration due to chronic smoking in young smokers was successfully demonstrated.

Chronic smoking increases the number of damaged endothelial cells in the blood circulatory system, resulting in morphological and functional changes within the endothelium [36,37,38] and causing higher risk of arteriosclerosis [39]. When the arteriolar vasomotor function is damaged, vasodilation and vasoconstriction response to blood CO_2_ changes during the respiration cycle decreases, leading to a decrease in Δ*Y_r_*. No significant differences in the MAP and heart rate between non-smokers and smokers were observed, indicating a comparable cardiac output for each group (Table 2). Note that individual changes in the heart rate do not influence the spectral analysis of the venous blood MR signal whenever the heart rate is lower than the value determined by the Nyquist frequency (1/(2TR)) used in the analysis (84 min^−1^ in this study). The Brinkman index, which is calculated as the product of the number of cigarettes per day and the number of years of smoking, was not correlated with the values of Δ*Y_r_* of smokers (r = 0.25). The amount of nicotine in a cigarette is not considered in the Brinkman index (although it may also influence the effect of chronic smoking on the arteriolar vasomotor function).

This study has some limitations. Reproducibility of the test results must be clarified. There are two hypotheses in our study, namely constant respiratory rhythm (15 per minute) and constant respiratory PaCO_2_ level changes. Changes in the rhythm and depth of breathing affect the results. The practice of breathing by monitoring the rhythm and PetCO_2_ may help improve the reproducibility. Although acute smoking decreases Y due to the increase in carboxyhemoglobin (HbCO) [40], we used the same Y value (0.66) in Equation (1) for both non-smokers and smokers. Since the amount of HbCO is normally in the range of 4–6% for smokers and 1–2% for non-smokers [40], Y would be lower in smokers. Lowering Y decreases Δ*Y_r_* in Equation (1). Therefore, Δ*Y_r_* of smokers would further be lower than that of non-smokers. Hct would also influence the results since C in Equation (1) increases with an increase in Hct [35]. Therefore, evidence that Hct in smokers is larger than that in non-smokers at the ages in 20 to 32 [41] indicates a further decrease in Δ*Y_r_* for smokers. Both a Y decrease and an Hct increase in smokers lead to further lowering of Δ*Y_r_*, and hence the influence of Y and Hct does not jeopardize our conclusion. Furthermore, Y decreases with aging (decrease 0.014 in a decade) and gender difference of Y exists (0.021 higher in females) [42]. Our subjects were in their 20s, however, and we used the value of Y (0.66) from the previous study whose subjects (three females and nine males) were at the age of 33 ± 6 [34]; the age difference is roughly a decade. Even taking account into this age difference and the subjects’ gender difference, average Y would be almost the same value (0.67) as we used. Y (0.66 ± 0.03, *n* = 12) and Hct (0.45 ± 0.02, *n* = 18), hence C in Equation (1), varies individually, and this variation leads to 10 % variation in Δ*Y_r_* estimated by using the law of propagation of error; the obtained Δ*Y_r_* variation (57% for non-smokers, 77% for smokers, *n* = 10, respectively) in our study is quite larger than the estimated variation due to individual Y and Hct. To improve the measurement accuracy of Δ*Y_r_*, monitoring breath depth (PetCO_2_) may help it. Since our method is based on the ideal laminar-like blood flow through the imaging length (maximal slice thickness: 15 mm) of the superior sagittal sinus, the measurement method should also be improved addressing non-ideal cases that may happen. The temporal measurement method of Y in superior sagittal sinus using MRI [43] may be promising for the measurement of Δ*Y_r_*. However, countermeasures against the harmonics of the cardiac pulsation component as in our method are needed.

In this study, we analyzed the spontaneous changes in the MR time series signals of cerebral venous blood and calculated the respiration-induced venous oxygenation fluctuation, which reflects the arteriolar vasomotor function. A significant decrease in the value was clearly observed in young smokers when compared with that of non-smokers, demonstrating degeneration in the arteriolar vasomotor function due to chronic smoking.

## 5. Conclusions

By analyzing the spontaneous changes in the MR time series signals of cerebral venous blood, a significant decrease in the values of respiration-induced venous oxygenation fluctuation in young smokers when compared with non-smokers was observed, demonstrating degeneration in the arteriolar vasomotor function due to chronic smoking. This non-invasive method represents a new MRI technique to evaluate the arteriolar vasomotor function.

## Figures and Tables

**Table 1:** Status of smokers.

Smokers	Number of Cigarettes Per Day	Duration of Smoking [Years]	Brinkman Index
1	10	4	40
2	10	4	40
3	10	6	60
4	10	7	70
5	9	3	27
6	15	3.5	52.5
7	11.5	5	57.5
8	10	6	60
9	10	4	40
10	12.5	3	37.5
Mean ± SD	10.8 ± 1.7	4.6 ± 1.3	48.5 ± 12.7

Brinkman index = number of cigarettes per day × duration of smoking. SD, standard deviation. See Table S2 in Supplementary Materials for further details.

**Table 2:** Results of physiological parameters.

Physiological Parameters	Non-Smokers	Smokers
PetCO_2_ [mmHg]	32.7 ± 5.0	33.8 ± 4.5
MAP [mmHg]	81 ± 4	81 ± 9
Heart rate [min^−1^]	67 ± 8	61 ± 10

Mean ± SD. PetCO_2_: end-tidal partial pressure of CO_2_. MAP: mean arterial pressure. See Tables S1 and S2 in Supplementary Materials for further details.

## Data Availability

Data are available within the article or its Supplementary Materials.

## Supplementary Materials

The following supporting information can be downloaded at: https://www.mdpi.com/article/10.3390/tomography8020055/s1. Table S1: Data of non-smokers; Table S2: Data of smokers.
